# Comprehensive Physical Assessment in an Older Adult Inpatient Psychiatric Ward – A Pilot Project

**DOI:** 10.1192/bjo.2025.10417

**Published:** 2025-06-20

**Authors:** Jhon Miguel Vecida, Matthew Francis, Folakemi Oladinni, India Corrin, Eleanor Cook

**Affiliations:** Southwest London and St George’s Mental Health NHS Trust, London, United Kingdom

## Abstract

**Aims:** To improve the assessment and management of physical health comorbidities for patients admitted to Crocus Ward, Springfield University Hospital.

**Methods:** The Old Age PHA template was devised using the guidance from the RCPsych report 'Caring for the whole person and incorporated elements of the 'Comprehensive Geriatric Assessment Tool’ used in primary care and the Old Persons Adult Liaison (OPAL) team at St George’s Hospital, London.

In performing the assessment, clinicians gather information from multiple sources: patient interviews, collateral histories, and reviews of GP and hospital records, and recent investigations.

The following information was obtained: Past Medical History, smoking, alcohol, substance use, vision, hearing, oral health, bladder and bowel function, pain and falls assessment, physical examination, investigation results, risk calculator scores, Baseline 4AT score, Medication/Polypharmacy Review, STOPP/START tool, Anticholinergic burden (ACB) calculator result.

Using the collected information, clinicians develop tailored care plans and recommendations to address identified physical health concerns.

**Results:** From May 2024, all new admissions underwent a Comprehensive Physical Assessment, with the report, including care plans and recommendations, uploaded to their electronic records.

**Conclusion:** The comprehensive physical assessment has significantly improved patient care by enabling early identification of physical health issues and potential risks. It facilitates personalised care plans and serves as a valuable reference for the multidisciplinary team. This approach addresses immediate health concerns and contributes to better long-term outcomes. The project underscores the importance of integrating physical and mental health care in older adult psychiatric settings.

